# AB0 blood group and prognosis in patients with pancreatic cancer

**DOI:** 10.1186/1471-2407-12-319

**Published:** 2012-07-28

**Authors:** Nuh N Rahbari, Ulrich Bork, Ulf Hinz, Albrecht Leo, Johanna Kirchberg, Moritz Koch, Markus W Büchler, Jürgen Weitz

**Affiliations:** 1 Department of General, Visceral and Transplant Surgery, University of Heidelberg, Im Neuenheimer Feld, Heidelberg, 110 69120, Germany; 2 Institute for Clinical Transfusion Medicine and Cell Therapy Heidelberg, University of Heidelberg, Heidelberg, Germany

## Abstract

**Background:**

Although blood group 0 is associated with a reduced risk of pancreatic cancer, little is known about the role of AB0 blood group antigens in disease progression. We assessed the prognostic relevance of AB0 blood status in a large cohort of patients with resected pancreatic cancer.

**Methods:**

A total of 627 patients, who underwent resection for pancreatic ductal adenocarcinoma between October 2001 and December 2008 were enrolled. The relationship between AB0 blood group status and outcome was analyzed using univariate and multivariate Cox regression analyses.

**Results:**

In patients with pancreatic cancer the incidence of blood group 0 (31%) was lower compared to 13.044 patients without pancreatic cancer (38%) (p = 0.0005). There were no significant differences in clinicopathologic characteristics among patients with different AB0 blood groups. The 3-year and 5-year overall survival rates were 29% and 14%. On univariate analysis AB0 blood group status did not correlate with survival (p = 0.39). Multivariate analysis, however, revealed a favorable and independent impact of blood group 0 on survival (Hazard ratio 0.78; 95% confidence interval 0.62 – 0.99; p = 0.037).

**Conclusion:**

AB0 blood group status is associated independently with the prognosis of patients with resected pancreatic cancer.

## Background

Pancreatic cancer has a dismal prognosis with an overall 5-year survival rate of < 5% making it the 4^th^ leading cause of cancer related mortality in western countries [1]. Although various highly penetrant familial syndromes have been identified, most underlying genetic risk factors for sporadic pancreatic cancer remain elusive [2]. There is, however, substantial evidence of an association between the risk of developing gastrointestinal cancers and AB0 blood group status [3-6]. A recent genome-wide association study confirmed variants in the AB0 locus to be associated with pancreatic cancer [7]. Analyses of large, independent populations have confirmed a significantly lower risk of developing pancreatic cancer in people with blood group 0 [8-11], whereas a recent study did not find a significant association between AB0 blood group status and the incidence of pancreatic cancer [12].

The reduced incidence of pancreatic cancer among patients with blood group 0 has raised the question, whether AB0 blood group status also correlates with the outcome of patients who actually develop this disease. To date, only two studies have correlated AB0 blood group status with survival of pancreatic cancer patients and their results are inconsistent [10,13]. The importance of blood group antigens for disease progression in pancreatic cancer is underlined by studies that demonstrated a prognostic value of Carbohydrate 19–9 (CA19-9) in patients with resectable [14-16] and advanced disease [17,18]. CA19-9 is the sialylated Lewis a (sLe^a^) blood group antigen and was first described by Koprowski et al. in 1979 [19,20]. Serum levels of CA19-9 are elevated in 70-80% of pancreatic cancer patients [21-23] and are used routinely to monitor the course of the disease [24].

The Lewis blood group antigens form terminal carbohydrate structures on cellular surfaces [25]. The molecular structure of the Lewis antigens is related to that of the AB0 blood group antigens A, B and 0. Their biosynthesis proceeds from common precursors and involves stepwise addition of monosaccharides catalyzed by glycosyltransferases [26]. Despite the structural relationship of AB0 and Lewis blood group antigens and their known significance for development and progression of pancreatic cancer, respectively, little is known about the impact of AB0 blood group status on survival of patients with pancreatic cancer.

It was the aim of the present study to assess, if AB0 blood group 0 is associated with a favorable prognosis in a cohort of 627 patients with pancreatic cancer who underwent potentially curative resection.

### Methods

Patients with pancreatic cancer who underwent surgery at the Department of General, Visceral and Transplantation Surgery, University of Heidelberg between October 2001 and December 2008 were identified from a prospective database. Patients with histologically proven pancreatic ductal adenocarcinoma were eligible for analyses. Patients with R2 resection were excluded, as were patients who underwent palliative procedures without tumor resection (i.e. bilioenteric and/or gastroenteric bypass) or exploratory laparotomy only. Furthermore, we excluded patients who received neoadjuvant therapy.

The following information was obtained for the purpose of the present analysis: patient demographics, American Society of Anesthesiologists (ASA) physical status classification, preoperative serum 19–9 levels (normal range: < 37 U/l), surgical procedures performed, tumor size (T category), lymph node status (N category), margin status (R category), tumor grading, AB0/Rhesus blood group typing and duration of follow-up. The study was approved by the local ethics committee of the University of Heidelberg.

The standardized preoperative work-up included a physical examination, routine laboratory testing, chest X-ray and abdominal imaging by contrast-enhanced computed tomography (CT) or magnetic resonance imaging (MRI) and magnetic resonance cholangiopancreatography (MRCP).

All patients underwent exploratory laparotomy with the intent of curative tumor resection. Intraoperative findings of distant metastases and peritoneal carcinomatosis were considered as contraindications to resection, as was advanced involvement (i.e. > 180° of the circumference) of the superior mesenteric artery and/or celiac axis [27]. Isolated involvement of the superior mesenteric vein/portal vein was not a contraindication for resection [28]. Surgical resection was performed as described previously [29]. Pathological specimens were processed using a standardized protocol [30]. R1 resection was defined, if the distance of the tumor from the resection margin was ≤ 1 mm.

After surgery, patients were followed at our outpatient clinic and the European Pancreas Center (EPC). Follow-up visits were scheduled every three months in the first two years and every six months thereafter. A clinical examination, abdominal ultrasound and routine laboratory testing with evaluation of CA19-9 levels were carried out at each follow-up visit. A CT scan was performed at three months postoperatively and every 6 months thereafter. We contacted the primary care physicians to obtain follow-up information on those patients who were not treated at our institution postoperatively.

Adjuvant therapy with either 5-fluorouracil (5-FU) or gemcitabine was recommended to all patients who were able to tolerate postoperative chemotherapy regardless of margin status or tumor stage.

### Statistical analyses

Categorical data were expressed as absolute and relative frequencies. Continuous data were categorized and presented as absolute and relative frequencies, too. Comparisons of categorical variables among the blood groups were performed using the *χ*^*2*^-test, if appropriate, or the Fisher's exact test. The distribution of the blood groups in the study group and the control group was compared using the *χ*^*2*^-test. Overall survival was calculated from the date of surgery to the date of death due to any cause or the date of last follow-up information. Survival curves were constructed according to the Kaplan-Meier method and compared using the log-rank test. Univariate survival analyses were performed based on the Cox proportional hazards regression methodology to identify individual risk factors related to long-term survival. Variables that were found to have a significant association with survival on univariate analyses were included in the multivariate models together with the blood group status. As a revised protocol for the analysis of pathological specimens was introduced in 2005 and markedly changed the incidence of R1 diagnoses, the R stage was not included in the multivariate models. Multivariate analyses of survival outcomes were done using Cox proportional hazards regression analyses. Results of the Cox regression analyses were reported with hazard ratio (HR) together with the corresponding 95% confidence intervals (CI). A *P* value of less than 0.05 was considered to indicate a statistically significant difference. All *P* values were two-sided. Calculations were performed using the SAS software package (SAS™ Version 9.1., SAS Institute Inc., Cary, USA).

## Results

A total of 627 patients with pancreatic ductal adenocarcinoma were eligible for final analyses (Table 1). There were 341 (54%) men and 286 (46%) women. Some 197 (31%) patients were aged ≥ 70 years. The vast majority of patients had T3 tumors (n = 597; 95%). Lymph node involvement was diagnosed in 515 (82%) patients. On final pathological examination tumors were well (G1), moderately (G2) and poorly differentiated (G3) in 19 (3%), 424 (68%) and 180 (29%) patients, respectively. Negative margins were achieved in 333 (53%) patients. Preoperative CA 19–9 levels were within the normal range in 130 (22%) patients, whereas 457 (78%) patients had elevated CA 19–9 levels. Some 521 (83%) patients were Rhesus D positive and 106 (17%) patients Rhesus D negative.

**Table 1:** Clinicopathologic characteristics of 627 patients with R0/1 resection for ductal adenocarcinoma of the pancreas

***Parameter***	***Total***	***Blood group A***	***Blood group AB***	***Blood group B***	***Blood group O***	***P-value***
n	627	328	30	73	196	
Age [years]						0.58
<70	430 (69%)	224 (68%)	24 (80%)	50 (68%)	132 (67%)	
≥70	197 (31%)	104 (32%)	6 (20%)	23 (32%)	64 (33%)	
Sex						0.98
Male	341 (54%)	177 (54%)	17 (57%)	41 (56%)	106 (54%)	
Female	286 (46%)	151 (46%)	13 (43%)	32 (44%)	90 (46%)	
Procedure						0.16
Pancreatico-duodenectomy	443 (71%)	242 (74%)	20 (67%)	50 (68%)	131 (67%)	
Distal pancreatectomy	108 (17%)	49 (15%)	9 (30%)	15 (21%)	35 (18%)	
Total pancreatectomy	76 (12%)	37 (11%)	1 (3%)	8 (11%)	30 (15%)	
Tumor size						0.89
pT0/1	9 (1%)	5 (2%)	1 (3%)	1 (1%)	2 (1%)	
pT2	10 (2%)	6 (2%)	1 (3%)	1 (1%)	2 (1%)	
pT3	597 (95%)	310 (94%)	28 (93%)	70 (96%)	189 (96%)	
pT4	11 (2%)	7 (2%)	0 (0%)	1 (1%)	3 (2%)	
Lymph node status						0.37
negative	112 (18%)	57 (17%)	9 (30%)	13 (18%)	33 (17%)	
positive	515 (82%)	271 (83%)	21 (70%)	60 (82%)	163 (83%)	
Grading						0.68
G1	19 (3%)	9 (3%)	2 (7%)	2 (3%)	6 (3%)	
G2	424 (68%)	224 (69%)	17 (57%)	47 (65%)	136 (70%)	
G3	180 (29%)	93 (28%)	11 (37%9	23 (32%)	53 (27%)	
missing values	4					
Resection margin						0.56
negative	333 (53%)	177 (55%)	18 (60%)	34 (47%)	104 (53%)	
positive	290 (47%)	147 (45%)	12 (40%)	39 (53%)	92 (47%)	
missing values	4					
CA 19–9 [U/ml]						0.43
<37	130 (22%)	34 (19%)	20 (29%)	6 (21%)	70 (23%)	
≥37	457 (78%)	145 (81%)	50 (71%)	23 (79%)	239 (77%)	
missing values	40					
Rhesus factor						0.40
positive	521 (83%)	267 (81%)	28 (93%)	62 (85%)	164 (84%)	
negative	106 (17%)	61 (19%)	2 (7%)	11 (15%)	32 (16%)	

About half of the patients had blood group A (n = 328; 52%). The number of patients with blood group AB, B and 0 was 30 (5%), 73 (12%) and 196 (31%), respectively. To evaluate, if this distribution and in particular the incidence of blood group 0 differed from that of a patient cohort without pancreatic cancer, we enrolled a control group of 13.044 patients who were treated at the Department of Surgery, University of Heidelberg for diagnoses other than pancreatic cancer during the same period of time (October 2001 until December 2008). In these patients, blood group 0, A, AB and B were present in 4.972 (38%), 5.823 (45%), 677 (5%) and 1.572 (12%) patients, respectively. The difference in the distribution of AB0 blood groups compared to patients with pancreatic cancer was statistically significant (p = 0.0006) and primarily caused by a lower incidence of blood group 0 vs. blood group A/B/AB compared to patients without pancreatic cancer (p = 0.0005).

There were no statistically significant associations between the AB0 blood group and the assessed clinicopathologic variables (Table 1). In particular, there was no significant association between the AB0 blood group status and CA 19–9 levels (p = 0.43). The proportion of patients with elevated preoperative CA19-9 levels was highest in patients with blood group 0. However, the difference in normal vs. elevated preoperative CA19-9 levels in patients with blood group 0 (19% vs. 81%) vs. A/B/AB (24% vs. 76%) did not reach statistical significance (p = 0.23).

Patients alive were followed for a median period of 26 months (range: 1–92 months). During the follow-up 412 (66%) patients died. The median overall survival was 22 months, the 3-year and 5-year overall survival rates were 29% and 14%, respectively. Seven patients (1%) were lost to follow-up.

**Figure 1:**
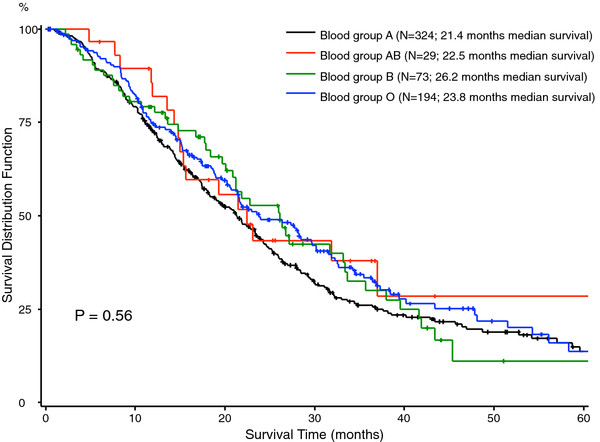
Overall survival in 620 patients with pancreatic ductal adenocarcinoma stratified for AB0 blood group status.

The association of clinicopathologic factors with patients’ survival was first evaluated on univariate analyses (Table 2). The factors age (p < 0.0001), lymph node status (P = 0.0077), grading of the tumor (p < 0.0001) and the resection margin (p = 0.025) correlated significantly with overall survival. Furthermore, patients with elevated preoperative CA 19–9 levels (i.e. values above the normal range) had significantly worse long-term outcome on univariate analyses (p = 0.0002). There was no significant difference in survival among the different AB0 blood groups on univariate analyses (p = 0.39) (Figure 1). Similarly, there was no survival advantage in the univariate analysis comparing patients with blood group 0 to the cohort of patients with blood group A/B/AB (p = 0.23). Patients’ Rhesus factor D status had no impact on survival (p = 0.74).

**Table 2:** Results from univariate Cox regression analyses of clinicopathologic parameters including blood group status on overall survival

***Parameter***	***Category***	***N***	***Events***	***HR***	***95% CI***	***P-value***
Age	<70 years	426	273	1		<0.0001
	≥70 years	194	139	1.54	1.26 – 1.89	
Sex	Male	336	235	1		0.21
	Female	284	177	0.88	0.73 – 1.07	
Tumor size	pT1/2	19	13	0.70	0.40 – 1.21	0.19
	pT3/4	601	399	1		
Lymph node status	Negative	111	68	1		0.0077
	Positive	509	344	1.42	1.10 – 1.85	
Procedure	Pancreatico-duodenectomy	440	302	1		0.06
	Distal pancreatectomy	106	58	0.82	0.62 – 1.09	
	Total pancreatectomy	74	52	1.33	0.99 – 1.78	
Grading	G1	19	9	0.38	0.20 – 0.74	<0.0001
	G2	419	267	1		
	G3	178	132	1.51	1.23 – 1.87	
Resection margin	Negative	329	232	1		0.0251
	Positive	287	177	1.25	1.03 – 1.53	
CA 19–9 [U/ml]	<37	127	68	1		0.0002
	≥37	453	316	1.65	1.27 – 2.14	
Blood group	A	324	228	1		0.39
	AB	29	17	0.76	0.47 – 1.25	
	B	73	46	0.89	0.65 – 1.23	
	O	194	121	0.85	0.68 – 1.06	
Blood group	A/B/AB	426	291	1		0.23
	O	194	121	0.88	0.71 – 1.09	
Rhesus factor	Positive	514	340	1		0.74
	Negative	106	72	0.96	0.74 – 1.24	

Analyses include 620 patients with R0/1 pancreatic resection for ductal adenocarcinoma of the pancreas (7 patients with incomplete follow-up excluded). HR, hazard ratio; CI; confidence interval.

On multivariate analyses that included all clinicopathologic factors with significant findings on univariate survival analyses well-differentiated tumors (G1) were associated with a favorable prognosis, whereas poorly differentiated tumors (G3), lymph node involvement, age ≥ 70 years and preoperatively elevated CA 19–9 levels were revealed as independent predictors of poor survival. In the primary multivariate model the individual AB0 blood groups were included using the most frequent blood group A as reference category. This multivariate analysis confirmed the a priori hypothesis that blood group 0 is associated with a favorable prognosis (HR 0.78; 95% CI 0.62 – 0.99; p = 0.037), whereas there was no significant survival benefit for patients with blood group AB (HR 0.82; 95% CI 0.49 – 1.38; p = 0.46) and blood group B (HR 0.81; 95% CI 0.58 – 1.14; p = 0.23) (Table 3). We, moreover, assessed the prognostic value of blood group 0 compared to non-0 blood groups in a second multivariate including patients’ blood group categorized as blood group 0 vs. the alternative blood groups A/B/AB. In this analysis the survival advantage for patients with blood group 0 just failed to reach statistical significance (HR 0.81; 95% CI 0.65 – 1.02; p = 0.078) (Table 4).

**Table 3:** Multivariate Cox regression analyses of prognostic factors on overall survival of 576 patients with R0/1 pancreatic resection for ductal adenocarcinoma of the pancreas

***Parameter***	***Category***	***HR***	***95% CI***	***P-value***
Grading	G1	0.44	0.22 – 0.86	0.017
	G2	1		
	G3	1.51	1.21 – 1.88	0.0003
Lymph node status	Negative	1		
	Positive	1.52	1.15 – 2.00	0.0031
Age [years]	< 70	1		
	≥ 70	1.44	1.15 – 1.79	0.0012
CA 19–9 [U/ml]	< 37	1		
	≥ 37	1.54	1.18 – 2.00	0.0016
AB0 blood group	A	1		
	0	0.78	0.62 – 0.99	0.037
	AB	0.82	0.49 – 1.38	0.46
	B	0.81	0.58 – 1.14	0.23

A total of 44 patients with missing values were excluded (Likelihood-Ratio Test: p < 0.0001).

HR = hazard ratio, CI = confidence interval.

**Table 4:** Multivariate Cox regression analysis of prognostic factors for overall survival in 576 patients with R0/1 pancreatic resection for ductal adenocarcinoma of the pancreas

***Parameter***	***Category***	***HR***	***95% CI***	***P-value***
Grading	G1	0.42	0.21 – 0.82	0.011
	G2	1		
	G3	1.48	1.18 – 1.84	0.0005
lymph node status	Negative	1		
	Positive	1.52	1.15 – 1.99	0.0031
Age [years]	< 70	1		
	≥ 70	1.43	1.15 – 1.78	0.0011
CA 19–9 [U/ml]	< 37	1		
	≥ 37	1.53	1.75 – 2.00	0.0016
AB0 blood group	A/B/AB	1		
	0	0.81	0.65 – 1.02	0.079

A total of 44 patients with missing values were excluded (Likelihood-Ratio Test: p < 0.0001).

CI = confidence interval, HR = hazard ratio.

Finally, we investigated the interaction of AB0 blood group status and preoperative CA 19–9 expression on patients’ prognosis. Patients were stratified into four groups according to the AB0 blood group (0 vs. A/B/AB) and preoperative CA19-9 levels (< 37 μg/l vs. ≥ 37 μg/l). The multivariate analysis including these combination terms confirmed the additional prognostic value of AB0 blood group status to CA 19–9 expression. In comparison to patients with preoperatively elevated CA 19–9 levels and blood group A/AB/B patients with normal CA 19–9 levels and blood group 0 had a significant survival advantage (HR 0.43; 0.25 – 0.74; p = 0.002), whereas there was only a moderate survival benefit for patients who had normal CA 19–9 levels and blood group A/AB/B (HR 0.71; 0.52 – 0.96; p = 0.028) (Table 5). These analyses confirmed the more pronounced impact of CA 19–9 expression on patients’ prognosis and, moreover, suggested a lack of prognostic relevance of the AB0 blood group status in patients with elevated CA 19–9 levels (HR 1.15; 0.90 – 1.47; p = 0.24).

**Table 5:** Final model of the multivariate Cox regression analysis of prognostic factors on overall survival including the interaction term CA19-9 and AB0 blood group of 576 patients with R0/1 pancreatic resection for ductal adenocarcinoma of the pancreas

***Parameter***	***Category***	***HR***	***95% CI***	***P-value***
				
Grading	G1	0.41	0.21 – 0.81	0.01
	G2	1		
	G3	1.48	1.19 – 1.84	0.0004
Lymph node status	Negative	1		
	Positive	1.52	1.16 – 2.01	0.0026
Age	< 70 years	1		
	≥ 70 years	1.42	1.14 – 1.77	0.003
CA 19–9 [U/ml] / AB0 blood group	≥ 37 / A/AB/B	1		
	< 37 / 0	0.43	0.26 – 0.74	0.002
	≥ 37 / 0	1.15	0.90 – 1.47	0.24
	< 37 / A/AB/B	0.71	0.52 – 0.96	0.028

A total of 44 patients with missing values were excluded (Likelihood-Ratio Test: p < 0.0001).

HR = hazard ratio, CI = confidence interval.

## Discussion

Recently published studies demonstrated the risk for pancreatic cancer of being significantly lower in patients with blood group 0 [8-11]. Owing to these data, we hypothesized that the genetic background associated with this blood group might predispose those patients who actually develop pancreatic cancer to a more favorable prognosis. The available evidence on the prognostic value of AB0 blood group in patients with pancreatic cancer has been conflicting. In a study on 417 patients Dandona et al. confirmed the increased risk for the development of pancreatic cancer in patients with blood groups others than 0, but did not observe a significant effect of AB0 blood group on overall survival [13]. Ben et al. also confirmed the reduced incidence of pancreatic cancer in patients with blood group 0 in their cohort study of 1431 Han Chinese patients [10]. Using univariate analysis these authors, moreover, reported a more favorable prognosis of patients with blood group 0 compared to patients with non-0 blood group in the subgroup of 316 patients who underwent potentially curative resection, whereas the survival between the individual blood groups did not differ significantly. Interestingly, in this study AB0 blood group did not correlate with survival in those patients who did not undergo potentially curative surgery.

The present study on 627 patients showed a favorable association of blood group 0 with survival after potentially curative resection for pancreatic cancer. In accordance with previous analyses differences in the survival of patients were not apparent on univariate analyses comparing the individual outcome of patients with one of the four AB0 blood groups [10,13]. However, multivariate analyses revealed blood group 0 as an independent predictor of long-term survival, though of less prognostic significance compared to CA 19–9 expression. To our knowledge this is the first analysis to evaluate the prognostic value of AB0 blood group status on multivariate analyses considering other well-known risk factors in pancreatic cancer and in particular CA 19–9 as member of the Lewis blood group family. The reasons, why this difference in survival is not evident on univariate analysis remain unclear at this stage. Even though the Kaplan-Meier plots already suggest improved survival of patients with blood group 0 compared to patients with blood group A, these data suggest that blood group 0 is associated with less aggressive biological features of disease. As our analyses on the association of AB0 blood group status with clinical and pathological variables of disease did not indicate any significant differences between individual blood group types these favorable tumor characteristics remain unknown and should be subject to further investigation.

The relationship between cell surface carbohydrate structures such as the AB0 and the Lewis blood group antigens and the biological behavior of tumors has been described previously [25,31]. While alterations of cellular adhesion, membrane signaling and cellular immune responses may play an important role, the exact mechanisms by which the expression of certain carbohydrate structures influence tumor progression remain incompletely understood. There is substantial evidence that tumor-promoting actions of CA19-9 (sLe^a^) are linked to its role as a ligand for the selectin family and thus to the ability of tumor cells to adhere to endothelial cells [32,33]. Preclinical studies showed that the expression of selectin ligands sLe^a^ and sLe^x^ on tumor cells was induced by hypoxia and increased the cellular adhesion of these selected clones to endothelial cells [34,35]. Subsequent *in vivo* studies demonstrated tumor angiogenesis to be significantly dependent upon E-selectin mediated cell adhesion of tumor cells expressing sLe^a^ and sLe^x^ to endothelial cells [36]. Furthermore, the adhesion of tumor cells via sLe^a^ and sLe^x^ to vascular endothelial cells may facilitate hematogenous metastasis [33]. Although several studies have demonstrated a prognostic value for the AB0 blood group antigens in various malignancies, the results of these studies are inconsistent suggesting that the biological role of ABH antigens may be disease-specific [37-40]. Experimental data on murine colon cancer cells suggest the expression of blood group antigens A and B to mediate the resistance to apoptosis which may facilitate the escape from immune surveillance [41].

As recently demonstrated, AB0 blood group status is, moreover, associated with serum levels of soluble intercellular adhesion molecule-1 (sICAM-1) [42], which is a member of the immunoglobulin superfamily of adhesion receptors and the soluble from of intercellular adhesion molecule-1 (sICAM-1) [43]. SICAM-1 is capable of inhibiting lymphocyte attachment to endothelial cells by binding to the ICAM ligands on circulating cells [44]. Levels of sICAM-1 are significantly decreased in patients with blood groups A and B (in particular blood group A) compared to patients with blood group 0 [42]. Decreased serum concentrations of sICAM have been implicated with various diseases, possibly via enhanced adhesion of leucocytes to endothelial surfaces and, by this, increased inflammatory conditions that in turn may promote tumor development and progression [45,46]. As some cancer calls use similar mechanisms for adhesion to endothelial cells and subsequent metastasis formation [47,48], the decreased sICAM levels in patients with non-0 blood groups may also promote metastatic spread of tumors. This may indeed help to explain the finding of our study showing blood group 0 to be associated with a favorable prognosis primarily in pancreatic cancer patients with normal CA19-9 levels. One might speculate that in patients with elevated CA19-9 levels tumor cell adhesion to endothelial cells is primarily mediated via sLe^a^ and E-selectin, whereas in patients with normal CA19-9 levels ICAM-mediated adhesion may compensate for the lack of sLe^a^. In the latter patients the interference of blood group 0 and the associated increased sICAM levels on endothelial cell adhesion of tumor cells may therefore become clinically relevant. However, further studies are required to investigate the molecular mechanisms by which AB0 blood group influences the course of disease in patients with pancreatic cancer.

## Conclusion

In conclusion, the results of the present analysis suggest a favorable influence of blood group 0 on survival of patients undergoing potentially curative resection for pancreatic cancer. To our knowledge, this study represents the largest analysis of the prognostic value of AB0 blood group status in patients with resected pancreatic cancer published so far and the first to utilize multivariate analyses considering other well-known prognosticators. While preoperative CA 19–9 expression was shown to be of stronger prognostic relevance, our findings of an independent and additional prognostic value of AB0 blood group status suggest that the prognostic influence of both blood group antigen families is mediated via distinct molecular mechanisms. Together with data showing the association of AB0 blood group status for development of pancreatic cancer, our results corroborate the significance of ABO blood group antigens for disease progression in pancreatic cancer. Subsequent studies are required to elucidate the underlying molecular mechanisms.

## Competing interests

The authors declare that they have no competing interests.

## Authors’ contributions

This study was designed by NNR, UB and JW. The article was written by NNR and UB. NNR, UB, AL, JK and MK were involved in data acquisition. UH performed the statistical analyses. UH, AL, JK, MK and MWB critically revised the manuscript. All authors have read and approved the manuscript.

## Pre-publication history

The pre-publication history for this paper can be accessed here:

http://www.biomedcentral.com/1471-2407/12/319/prepub
